# Mental health status of Chinese residents during the COVID-19 epidemic

**DOI:** 10.1186/s12888-020-02966-6

**Published:** 2020-12-03

**Authors:** Wen Jiang, Xuerong Liu, Jingxuan Zhang, Zhengzhi Feng

**Affiliations:** School of Psychology, Army Medical University, Chongqing, China

**Keywords:** COVID-19, Mental health, Depression, Anxiety, Negative cognitive processing bias

## Abstract

**Background:**

To investigate the mental health status of Chinese residents during the epidemic of COVID-19, as well as to identify the positive and negative factors and regulatory effect of negative cognitive processing bias on mental health.

**Methods:**

A total of 60,199 residents in China were surveyed via an internet-based survey containing a general questionnaire, such as the self-rating depression scale, the state anxiety inventory, and the negative cognitive processing bias questionnaire. An ordered multiple logistic regression analysis model was used to analyze the collected data.

**Results:**

The survey revealed mild, moderate, and severe depressive symptoms in 62.65, 11.33, and 6.14% participants, respectively, and mild, moderate, and severe anxiety symptoms in 33.21, 41.27, and 22.99% participants, respectively. Multiple logistic regression analysis showed that factors, such as female gender, being older than 55 years, high school education level, medical staff, marital conflicts, negative attention bias, rumination, and death growth rate, positively affected depression and anxiety symptoms. The good family functionality, democratic working atmosphere, and a myriad of social activities negatively affected the level of depressive and anxiety symptoms.

**Conclusion:**

Chinese residents exhibited a high prevalence of anxiety and depressive symptoms during the epidemic. Thus, psychological interventions should focus on the vulnerable groups, and cognitive training should focus on reducing the negative cognitive processing bias. This might be an effective way to alleviate the mental stress of the general public during the COVID-19 pandemic.

## Background

In late December 2019, the 2019 coronavirus disease (COVID-19) appeared in Wuhan City, Hubei Province, China [1]. On January 30, 2020, the World Health Organization (WHO) declared the COVID-19 epidemic as a public health emergency of international concern [2]. The number of confirmed cases and deaths is changing hourly and daily and can be tracked on the website of National Health Commission of China [3]. According to these numbers, the daily confirmed growth rate of cases and death growth rates reflect changes in the epidemic [4]. As of late February 2020, China had a total of nearly 80,000 confirmed cases and nearly 3000 deaths, thereby causing a large burden of morbidity and mortality.

As a result of the rapidly increasing numbers of confirmed cases and deaths, Chinese residents have been experiencing psychological problems, including anxiety and depression [5]. The severity of COVID-19 infection, the uncertainty of how to control the disease, and information overload, can raise concerns among the population [6]. In addition, with the implementation of isolation policy, social activities have been drastically decreased, and the psychological stress has increased [7], resulting in anger and loneliness [5]. The previous study has shown that such challenges and stresses may lead to common mental disorders [8]. Besides, cognitive factors may affect public mental health and influence anxiety and depression when facing the COVID-19 epidemic. The diathesis-stress theory states that the interaction between external life events and individuals’ internal lives leads to psychological problems [9]. The cognitive model of depression postulates that depression symptoms are maintained by negatively biased cognition, including negative attention bias, negative memory bias, and rumination [10–12]. The negative cognitive processing influences what people attend to, how they interpret new information, and what they remember later in time, thus exacerbating and sustaining the negative mood that typifies depressive episodes [13, 14]. Research conducted over the last 50 years supports this proposition [15, 16]. In addition, negative cognitive processing bias could also negatively predict an individual’s mental health [17]. Therefore, this study assumed that the public’s mental health was related to external factors such as epidemic information, work environment, family conditions, and social activities, and internal factors such as cognitive processing.

Thus far, studies on depression and anxiety during COVID-19 have primarily focused on the medical staff. Only a few studies have examined the mental health of ordinary residents. Some previous studies have pointed out that the psychological impact caused by public health emergencies, such as the severe acute respiratory syndrome (SARS) epidemic in China in 2003 [18] and the Middle East respiratory syndrome (MERS) epidemic in 2012 [19, 20], may last for a long time and may bring severe psychological trauma to the people [21].

This cross-sectional study explores the effect of various factors on residents’ mental health under stress during public health emergencies. It provides accurate decision-making reference to the government departments with respect to the mental health of normal people.

## Methods

### Participants

The current study used a snowball sampling approach to distribute questionnaires online in Mainland China between 23 and 29 February 2020. The questionnaires were distributed via WeChat, Tencent QQ, and other public platforms. When participants completed the questionnaire, they forwarded it to their own WeChat circle of friends or other public platforms to expand the sample size. Each IP address could only be used once. The inclusion criteria were as follows: (1) 18–65-years-old, (2) native Chinese residents able to complete the questionnaires on the cellphone or computer, (3) informed consent. The exclusion criteria were: (1) unable to read correctly or use a computer or cellphone to complete the questionnaires, (2) refused to participate in the research.

In order to control bias, the questionnaires were initially distributed in the same number in each provincial capital city. The daily sample size was based on a national survey experience, and the sample size was set to be more than 1500 [22].

A total of 66,152 questionnaires were returned. On February 26 and 27, only 5 individuals filled out the questionnaire. Therefore, the data of these 2 days were excluded. Five thousand nine hundred forty-eight questionnaires were excluded such due to missing data, incomplete information, or extreme data. After deleting these substandard responses, 60,199 valid questionnaires were analyzed, with an effective rate of 91%.

The current study was approved by the Ethics Committee of the First Affiliated Hospital of Chongqing Medical University, China. All participants confirmed the informed consent before answering the questionnaires.

### Measures

#### Self-compiled descriptive characteristics questionnaire

Demographic data, work environment, family conditions, and social activities were covered by 13 items, including gender, age, education level, occupation, marital status, family structure in childhood, whether the participant was an only child, parenting style in childhood, whether the participant lived with his parents until the age of 10, number of close friends, the collective atmosphere in work/school, the management style of work/school, and social activities of last 2 weeks. According to Baumrind’s research [23], parenting style in childhood was divided into authoritarian, neglectful, permissive, and democratic. The management style of work/school was divided into three most common types of leadership styles, i.e., autocratic, laissez-faire, and democratic, as defined by Kurt Lewin [24].

#### Depressive symptoms

The self-rating depression scale (SDS) [25] contained 20 items, and the design was based on the diagnostic criteria for depression. The subjects rated each item using a 4-point Likert scale based on how they have felt during the past several days. The SDS’s raw sum score was 20–80; however, the results were presented as the SDS index, which is obtained by expressing the raw score converted to a 100-points scale. The cut-off value of the SDS standard score was 53, 53–62 for mild depressive symptoms, 63–72 for moderate depressive symptoms, and > 73 with severe depressive symptoms according to the Chinese norm [26]. The Chinese version had good internal consistency reliability of the total scale (α = 0.86) [27]. The Cronbach’s α in the current study was 0.63.

#### Anxiety symptoms

The state anxiety inventory (SAI) is a scale from the state-trait anxiety inventory [28], containing 20 items to evaluate state anxiety under stress, using a 4-point Likert scale. The total score ranged from 20 to 80, according to the score boundaries: 20–39 without anxiety symptoms, 40–47 with mild anxiety symptoms, 48–54 with moderate anxiety symptoms, and 55 ~ 80 with severe anxiety symptoms. The Chinese version had good internal consistency reliability of the total scale (α = 0.91) [29]. The Cronbach’s α in the current study was 0.68.

#### Negative cognitive processing bias questionnaire (NCPBQ)

NCPBQ is a 16-item self-report measure in Chinese, used for assessing the negative attention bias, negative memory bias, and rumination, using a 4-point Likert scale (1 = not match; 4 = perfect match) [16]. An example of an item is “I always remember my mistakes clearly.” Higher total scores indicate negative cognitive processing bias. It had good internal consistency reliability of the total scale (α = 0.89) in college students’ normal population. The Cronbach’s α of the current study was 0.84, and that of negative attention bias, negative memory bias, and rumination was 0.78, 0.68, and 0.72, respectively.

### Confirmed growth rate and death growth rate

The confirmed growth rate was calculated as the ratio between the cumulative number of confirmed cases announced on the day and on the previous day to the cumulative number of confirmed cases announced on the previous day. The death growth rate was the ratio of the difference between the cumulative number of deaths announced on the day and on the previous day to the cumulative number of deaths announced on the previous day. The number of confirmed cases and deaths was provided by the National Health Commission of China [3].

### Depressed group and anxiety group

According to the SDS scoring criteria, the participants were divided into non-depressed, mild depressive symptoms, moderate depressive symptoms, and severe depressive symptom groups. They were also divided into non-anxiety, mild anxiety symptom, moderate anxiety symptom, and severe anxiety symptom groups according to the SAI scoring criteria.

### Data analysis

The data were analyzed using software SPSS 23.0 and SAS 9.4. Measurement data were expressed as mean ± standard deviation ($\overline{x}$ ± sd). The age data were divided down by the maximum age, with every ten years as an age group. Enumeration data were expressed by the number of people (%). Pearson’s correlation coefficient evaluated the correlation between the severities of depressive and anxiety symptoms, and *P* < 0.05 on double sides was considered to be statistically significant. The analysis of the correlations between characteristics (gender, age, education level, occupation, family structure in childhood, whether the participant was an only child, parenting style in childhood, whether the participant lived with his parents until the age of 10, number of close friends, the collective atmosphere in work/school, the management style of work/school, and social activities of last 2 weeks) and anxiety or depressive symptoms initially were assessed by the chi-square test. The correlations between negative cognitive processing bias (negative attention bias, negative memory bias, and rumination), confirmed growth rate, death growth rate, and anxiety or depressive symptoms were initially assessed by one-way analysis of variance (ANOVA). The variables with *P* < 0.05 were entered in the ordered multiple logistic regression analysis models by the stepwise method.

## Results

### General characteristics of the participants

The cohort included 34,418 (57.2%) females and 25,781 (42.8%) males among the 60,199 questionnaires, aged 18–65 (average: 34.66 ± 12.02)-years-old. The demographic characteristics of participants are shown in Table 1.

**Table 1:** Descriptive characteristics of the participants (*n* = 60,199)

Variables	Grouping	N (%)
Gender	Female	34,418 (57.17%)
	Male	25,781 (42.83%)
Age (years)	18–25	17,858 (29.66%)
	26–35	18,445 (30.64%)
	36–45	10,345 (17.18%)
	46–55	9864 (16.39%)
	56–65	3687 (6.12%)
Education level	Primary school	5585 (9.28%)
	Middle school	1733 (2.88%)
	High school	19,028 (31.61%)
	Bachelor’s degree	31,098 (51.66%)
	Master’s degree or above	2755 (4.58%)
Occupation	Worker	10,001 (16.61%)
	Farmer	3615 (6.01%)
	Soldier	5262 (8.74%)
	Medical staff	9153 (15.20%)
	Teacher	7396 (12.29%)
	Cadre	3777 (6.27%)
	White collar	12,106 (20.11%)
	Other	8889 (14.77%)
Family structure in childhood	Two parents	42,116 (69.96%)
	One parent	10,742 (17.84%)
	Other	7341 (12.19%)
Only child in the family	No	12,051 (20.02%)
	Yes	48,148 (79.98%)
Parenting style in childhood	Authoritarian	28,396 (47.17%)
	Neglectful	2546 (4.23%)
	Permissive	17,706 (29.41%)
	Democratic	11,551 (19.19%)
Living with parents before 10-years-old	Yes	34,581 (57.44%)
	No	25,618 (42.56%)
Number of close friends	None	6199 (10.30%)
	1–2	21,791 (36.20%)
	> 3	32,209 (53.50%)
The collective atmosphere in work/school	Peace and tranquility	19,135 (31.79%)
	Occasional quarrels	33,570 (55.77%)
	Frequent quarrels	7494 (12.45%)
The management style of work/school	Autocratic	45,270 (75.20%)
	Laissez-faire	2349 (3.90%)
	Democratic	12,580 (20.90%)
Marital status	Unmarried	12,530 (20.81%)
	Married	29,299 (48.67%)
	Divorced	10,652 (17.69%)
	Remarried	6265 (10.41%)
	Widowed	1453 (2.41%)
Social activities of last 2 weeks	0–2	22,790 (36.48%)
	3–5	17,003 (28.24%)
	6–8	8366 (13.90%)
	> 9	12,040 (20.00%)
Investigation date	February 23	9642 (16.02%)
	February 24	6598 (10.96%)
	February 25	6783 (11.27%)
	February 28	27,148 (45.10%)
	February 29	10,028 (16.66%)

### Confirmed growth rate and death growth rate

**Fig. 1:**
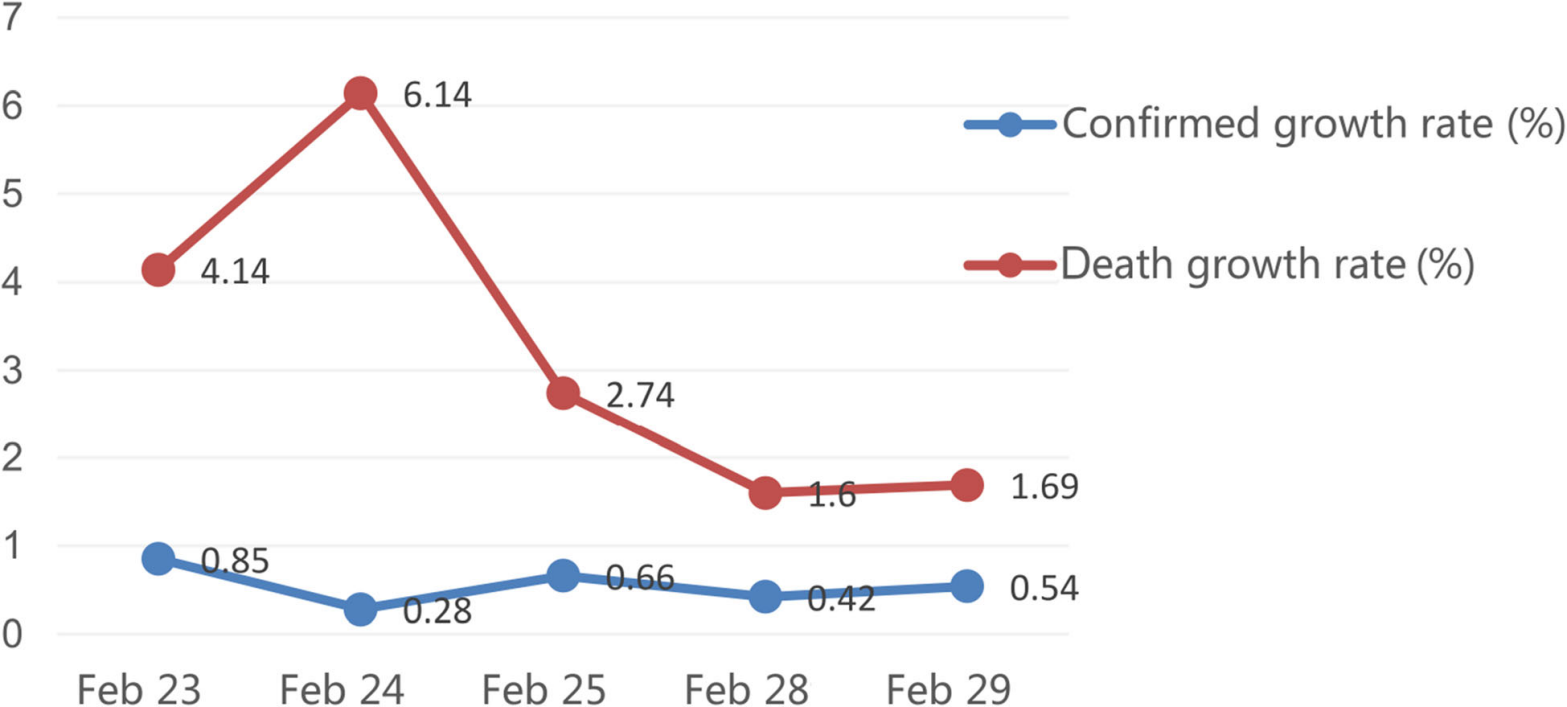
Confirmed growth rate and death growth rate of COVID-19 in China during the study

According to the cumulative numbers of confirmed cases and deaths of COVID-19 in China provided by the National Health Commission of the People’s Republic of China, we found that the confirmed growth rate fluctuated between 0.28 and 0.85%. The death growth rate fluctuated between 1.60 and 6.14% from February 23–29 (Fig. 1).

### Prevalence of anxiety and depressive symptoms

**Fig. 2:**
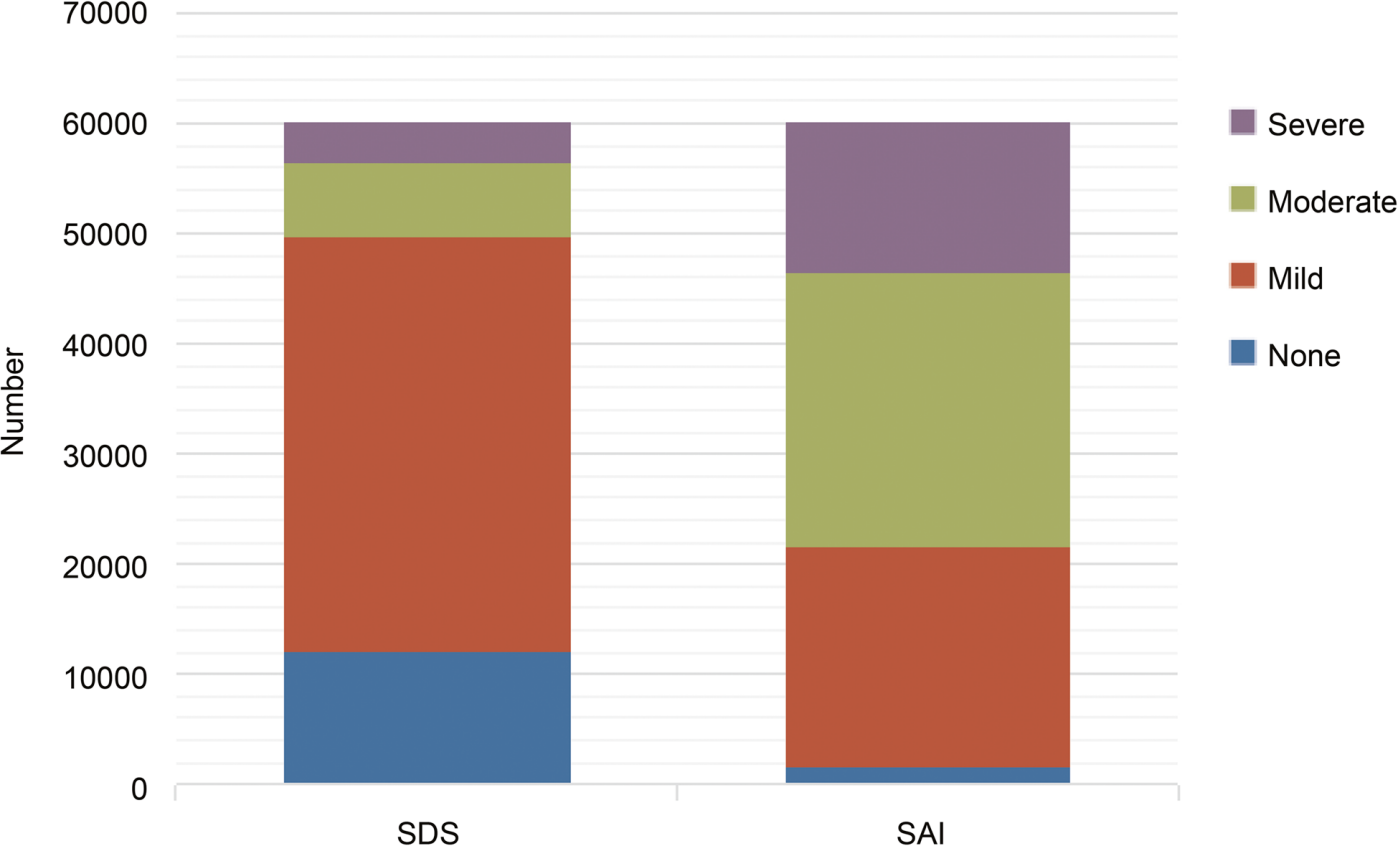
A number of people with different depression and anxiety degrees

The SDS standard score of all the participants was 58.31 ± 8.46 points; among them, 19.89% were non-depressed, 62.65% were mildly depressed, 11.33% were moderately depressed, and 6.14% were severely depressed. The participants’ SAI score was 51.52 ± 7.52 points; 2.53% were non-anxious, 33.21% were mildly anxious, 41.27% were moderately anxious, and 22.99% were severely anxious (Fig. 2). The correlation analysis showed a significant positive correlation between the severity of depressive and anxiety symptoms (r = 0.33, *P* < 0.001).

### Analysis of related factors of anxiety and depressive symptoms

Single-factor chi-square test results showed significant differences in the ratio of different degrees of depressive symptom and the ratio of different degrees of anxiety symptom with respect to gender, age, education level, occupation, family structure in childhood, whether the participant was an only child in the family, parenting style in childhood, whether the participant lived with parents until the age of 10, a number of close friends, collective atmosphere in work/school, the management style of work/school, marital status, and social activities of last 2 weeks (*P* < 0.001). The one-way analysis of variance results showed significant differences in the scores of negative attention bias, negative memory bias, rumination, confirmed growth rate, and death growth rate associated with different degrees of depressive and anxiety symptoms (*P* < 0.001).

In the ordered multiple logistic regression model of depressive symptoms, all the factors were correlated with the severity of depressive symptoms, except for the following factors: being the only child in the family, education level at master’s degree or above, white-collar, neglectful or permissive parenting style in childhood. Negative attention bias, rumination, confirmed growth rate, and death growth rate had positive effects on the severity of depressive symptoms. In contrast, negative memory bias had a negative effect on the severity of depressive symptoms (Table 2). In the ordered multiple logistic regression model of anxiety symptoms, all the factors were correlated with the severity of anxiety, except the confirmed growth rate, teacher, other jobs, having 1–2 close friends, the laissez-faire management style of work/school. Negative attention bias, negative memory bias, rumination, and death growth rate had positive effects on the severity of anxiety symptoms (Table 3).

**Table 2:** Ordered multiple logistic regression analysis of depression-related factors

Factors	Non-depressed group (*n* = 11,971) N(%)/ M ± SD	Mild depressive symptom group (*n* = 37,713) N(%)/ M ± SD	Moderate depressive symptom group (*n* = 6819) N(%)/ M ± SD	Severe depressive symptom group (*n* = 3696) N(%)/ M ± SD	χ^2^	*P*	*B*	OR (95% CI)	*P*
Gender									
Female	6481 (18.8%)	21,714 (63.1%)	3999 (11.6%)	2224 (6.5%)	67.133	< 0.001	Referent	1.0	–
Male	5490 (21.3%)	15,999 (62.1%)	2820 (10.9%)	1472 (5.7%)			−0.307	0.736 (0.705–0.767)	< 0.001
Age (years)									
18–25	6163 (34.5%)	10,064 (56.4%)	1059 (5.9%)	572 (3.2%)	23,912.598	< 0.001	Referent	1.0	–
26–35	3456 (18.7%)	13,318 (72.2%)	1088 (5.9%)	583 (3.2%)			0.601	1.824 (1.735–1.917)	< 0.001
36–45	1112 (10.7%)	8227 (79.5%)	660 (6.4%)	346 (3.3%)			0.813	2.255 (2.128–2.389)	< 0.001
46–55	953 (9.7%)	5173 (52.4%)	342 6 (34.7%)	312 (3.2%)			1.825	6.204 (5.833–6.597)	< 0.001
56–65	287 (7.8%)	931 (25.3%)	586 (15.9%)	1883 (51.1%)			3.961	52.520 (48.416–56.971)	< 0.001
Education level									
Primary school	1356 (24.3%)	3312 (59.3%)	680 (12.2%)	237 (4.2%)	240.618	< 0.001	Referent	1.0	–
Middle school	253 (14.6%)	1188 (68.6%)	208 (12.0%)	84 (4.8%)			0.579	1.783 (1.558–2.041)	< 0.001
High school	3427 (18.0%)	12,083 (63.5%)	2197 (11.5%)	1321 (6.9%)			0.851	2.343 (2.196–2.500)	< 0.001
Bachelor’s degree	6273 (20.2%)	19,529 (62.8%)	3392 (10.9%)	1904 (6.1%)			0.607	1.834 (1.728–1.947)	< 0.001
Master’s degree or above	662 (24.0%)	1601 (58.1%)	342 (12.4%)	150 (5.4%)			–	–	> 0.05
Occupation									
Worker	1716 (17.2%)	6618 (66.2%)	1065 (10.6%)	602 (6.0%)	1260.539	< 0.001	Referent	1.0	–
Farmer	644 (17.8%)	2056 (56.9%)	618 (17.1%)	297 (8.2%)			0.118	1.125 (1.030–1.229)	0.009
Soldier	891 (16.9%)	3276 (62.3%)	780 (14.8%)	315 (6.0%)			0.243	1.275 (1.172–1.388)	< 0.001
Medical staff	2093 (22.9%)	5359 (58.5%)	973 (10.6%)	728 (8.0%)			0.294	1.341 (1.236–1.456)	< 0.001
Teacher	1336 (18.1%)	5121 (69.2%)	560 (7.6%)	379 (5.1%)			0.554	1.741 (1.622–1.868)	< 0.001
Cadre	1119 (29.6%)	2099 (55.6%)	417 (11.0%)	142 (3.8%)			−0.391	0.676 (0.616–0.742)	< 0.001
White collar	2912 (24.1%)	7156 (59.1%)	1200 (9.9%)	838 (6.9%)			–	–	> 0.05
Other	1260 (14.2%)	6028 (67.8%)	1206 (13.6%)	395 (4.4%)			0.238	1.269 (1.193–1.350)	< 0.001
Family structure in childhood									
Two parents	8897 (21.1%)	25,977 (61.7%)	4748 (11.3%)	2494 (5.9%)	156.974	< 0.001	Referent	1.0	–
One parent	1889 (17.6%)	6854 (63.8%)	1243 (11.6%)	756 (7.0%)			0.323	1.382 (1.301–1.468)	< 0.001
Other	1185 (16.1%)	4882 (66.5%)	828 (11.3%)	446 (6.1%)			0.448	1.566 (1.463–1.676)	< 0.001
Only child in the family									
No	1950 (16.2%)	7990 (66.3%)	1467 (12.2%)	644 (5.3%)	161.151	< 0.001	Referent	1.0	–
Yes	10,021 (20.8%)	29,723 (61.7%)	5352 (11.1%)	3052 (6.3%)			–	–	> 0.05
Parenting style in childhood									
Authoritarian	5723 (20.2%)	17,917 (63.1%)	2857 (10.1%)	1899 (6.7%)	139.812	< 0.001	Referent	1.0	–
Neglectful	577 (22.7%)	1550 (60.9%)	320 (12.6%)	99 (3.9%)			–	–	> 0.05
Permissive	3492 (19.7%)	10,949 (61.8%)	2211 (12.5%)	1054 (6.0%)			–	–	> 0.05
Democratic	2179 (18.9%)	7297 (63.2%)	1431 (12.4%)	644 (5.6%)			−0.192	0.825 (0.775–0.878)	< 0.001
Living with parents before 10 years old									
Yes	7036 (20.3%)	21,089 (61.0%)	4101 (11.9%)	2355 (6.8%)	124.316	< 0.001	Referent	1.0	–
No	4935 (19.3%)	16,624 (64.9%)	2718 (10.6%)	1341 (5.2%)			0.292	1.340 (1.279–1.403)	< 0.001
Number of close friends									
None	1402 (22.6%)	3437 (55.4%)	897 (14.5%)	463 (7.5%)	197.072	< 0.001	Referent	1.0	–
1–2	4413 (20.3%)	13,675 (62.8%)	2288 (10.5%)	1415 (6.5%)			0.533	1.705 (1.561–1.863)	< 0.001
> 3	6156 (19.1%)	20,601 (64.0%)	3634 (11.3%)	1818 (5.6%)			0.250	1.284 (1.182–1.394)	< 0.001
The collective atmosphere in work/school									
Peace and tranquility	3254 (17.0%)	12,078 (63.1%)	2576 (13.5%)	1227 (6.4%)	301.141	< 0.001	Referent	1.0	–
Occasional quarrels	7119 (21.2%)	21,177 (63.1%)	3384 (10.1%)	1890 (5.6%)			−0.132	0.876 (0.839–0.915)	< 0.001
Frequent quarrels	1598 (21.3%)	4458 (59.5%)	859 (11.5%)	579 (7.7%)			−0.410	0.664 (0.617–0.714)	< 0.001
The management style of work/school									
Autocratic	8670 (19.2%)	28,464 (62.9%)	5397 (11.9%)	2739 (6.1%)	139.059	< 0.001	Referent	1.0	–
Laissez-faire	553 (23.5%)	1506 (64.1%)	153 (6.5%)	137 (5.8%)			0.490	1.632 (1.481–1.797)	< 0.001
Democratic	2748 (21.8%)	7743 (61.6%)	1269 (10.1%)	820 (6.5%)			−0.086	0.917 (0.863–0.975)	0.005
Marital status									
Unmarried	2680 (21.4%)	7710 (61.5%)	1368 (10.9%)	772 (6.2%)	425.677	< 0.001	Referent	1.0	–
Married	6156 (21.0%)	18,280 (62.4%)	3137 (10.7%)	1726 (5.9%)			−0.212	0.809 (0.765–0.856)	< 0.001
Divorced	1540 (14.5%)	6806 (63.9%)	1639 (15.4%)	667 (6.3%)			0.273	1.313 (1.225–1.408)	< 0.001
Remarried	1312 (20.9%)	3963 (63.3%)	560 (8.9%)	430 (6.9%)			−0.160	0.852 (0.788–0.921)	< 0.001
Widowed	283 (19.5%)	954 (65.7%)	115 (7.9%)	101 (7.0%)			−0.347	0.707 (0.601–0.832)	< 0.001
Social activities of last 2 weeks									
0–2	4672 (20.5%)	14,057 (61.7%)	2553 (11.2%)	1508 (6.6%)	493.130	< 0.001	Referent	1.0	–
3–5	3132 (18.4%)	10,500 (61.8%)	2156 (12.7%)	1215 (7.1%)			0.388	1.474 (1.403–1.548)	< 0.001
6–8	1243 (14.9%)	5714 (68.3%)	1034 (12.4%)	375 (4.5%)			0.137	1.146 (1.077–1.221)	< 0.001
> 9	2924 (24.3%)	7442 (61.8%)	1076 (8.9%)	598 (5.0%)			−0.209	0.812 (0.767–0.859)	< 0.001
Negative attention bias	12.24 ± 3.36	12.3 ± 3.28	13.70 ± 3.65	13.17 ± 3.81	388.962	< 0.001	0.047	1.048 (1.042–1.054)	< 0.001
Negative memory bias	12.04 ± 3.13	12.04 ± 3.24	12.27 ± 3.20	12.90 ± 3.35	88.817	< 0.001	−0.012	0.988 (0.982–0.994)	< 0.001
Rumination	6.55 ± 2.37	6.62 ± 2.42	7.57 ± 2.58	7.14 ± 2.62	350.426	< 0.001	0.022	1.022 (1.014–1.030)	< 0.001
Confirmed growth rate	0.45 ± 0.18	0.53 ± 0.15	0.56 ± 0.20	0.59 ± 0.26	1130.900	< 0.001	1.938	6.946 (6.243–7.728)	< 0.001
Death growth rate	3.41 ± 2.01	2.06 ± 0.88	3.63 ± 1.59	4.40 ± 1.48	7363.433	< 0.001	0.025	1.0245 (1.014–1.037)	< 0.001

**Table 3:** Ordered multiple logistic regression analysis of anxiety-related factors

Factors	Non-anxiety group (*n* = 1522) N(%)/ M ± SD	Mild anxiety symptom group (*n* = 19,994) N(%)/ M ± SD	Moderate anxiety symptom group (*n* = 24,843) N(%)/ M ± SD	Severe anxiety symptom group (*n* = 13,840) N(%)/ M ± SD	χ^2^	*P*	*B*	OR (95% CI)	*P*
Gender									
Female	983 (2.9%)	10,448 (30.4%)	14,390 (41.8%)	8597 (25.0%)	375.489	< 0.001	Referent	1.0	–
Male	539 (2.1%)	9546 (37.0%)	10,453 (40.5%)	5243 (20.3%)			−0.541	0.582 (0.558–0.608)	< 0.001
Age (years)									
18–25	47 (0.3%)	9407 (52.7%)	4006 (22.4%)	4398 (24.6%)	25,371.421	< 0.001	Referent	1.0	–
26–35	1382 (7.5%)	8712 (47.2%)	4595 (24.9%)	3756 (20.4%)			−0.194	0.824 (0.786–0.863)	< 0.001
36–45	9 (0.1%)	1013 (9.8%)	7879 (76.2%)	1444 (14.0%)			1.151	3.162 (2.996–3.338)	< 0.001
46–55	17 (0.2%)	659 (6.7%)	7756 (78.6%)	1432 (14.5%)			1.177	3.244 (3.072–3.425)	< 0.001
56–65	67 (1.8%)	203 (5.5%)	607 (16.5%)	2810 (76.2%)			3.16	23.664 (21.600–25.925)	< 0.001
Education level									
Primary school	397 (7.1%)	2133 (38.2%)	2142 (38.4%)	913 (16.3%)	1857.616	< 0.001	Referent	1.0	–
Middle school	19 (1.1%)	550 (31.7%)	984 (56.8%)	180 (10.4%)			1.497	4.470 (3.898–5.127)	< 0.001
High school	453 (2.4%)	4714 (24.8%)	8969 (47.1%)	4892 (25.7%)			1.979	7.237 (6.722–7.791)	< 0.001
Bachelor’s degree	625 (2.0%)	11,609 (37.3%)	11,555 (37.2%)	7309 (23.5%)			1.534	4.635 (4.313–4.981)	< 0.001
Master’s degree or above	28 (1.0%)	988 (35.9%)	1193 (43.3%)	546 (19.8%)			0.633	1.883 (1.688–2.100)	< 0.001
Occupation									
Worker	124 (1.2%)	2493 (24.9%)	5383 (53.8%)	2001 (20.0%)	3835.299	< 0.001	Referent	1.0	–
Farmer	59 (1.6%)	814 (22.5%)	1392 (38.5%)	1350 (37.3%)			0.440	1.552 (1.429–1.686)	< 0.001
Soldier	50 (1.0%)	1179 (22.4%)	2932 (55.7%)	1101 (20.9%)			0.177	1.194 (1.109–1.286)	0.005
Medical staff	375 (4.1%)	2421 (26.5%)	3400 (37.1%)	2957 (32.3%)			1.059	2.883 (2.670–3.113)	< 0.001
Teacher	76 (1.0%)	3403 (46.0%)	2545 (34.4%)	1372 (18.6%)			–	–	> 0.05
Cadre	71 (1.9%)	1576 (41.7%)	1525 (40.4%)	605 (16.0%)			−0.394	0.675 (0.618–0.737)	< 0.001
White collar	653 (5.4%)	4478 (37.0%)	4297 (35.5%)	2678 (22.1%)			0.276	1.317 (1.245–1.394)	< 0.001
Other	114 (1.3%)	3630 (40.8%)	3369 (37.9%)	1776 (20.0%)			–	–	> 0.05
Family structure in childhood									
Two parents	968 (2.3%)	14,648 (34.8%)	17,504 (41.6%)	8996 (21.4%)	525.993	< 0.001	Referent	1.0	–
One parent	447 (4.2%)	2896 (27.0%)	4306 (40.1%)	3093 (28.8%)			−0.146	0.864 (0.814–0.917)	< 0.001
Other	107 (1.5%)	2450 (33.4%)	3033 (41.3%)	1751 (23.9%)			0.560	1.750 (1.639–1.869)	< 0.001
Only child in the family									
No	285 (2.4%)	4200 (34.9%)	5304 (44.0%)	2262 (18.8%)	158.001	< 0.001	Referent	1.0	–
Yes	1237 (2.6%)	15,794 (32.8%)	19,539 (40.6%)	11,578 (24.0%)			0.075	1.078 (1.018–1.141)	0.010
Parenting style in childhood									
Authoritarian	895 (3.2%)	10,439 (36.8%)	10,027 (35.3%)	7035 (24.8%)	1902.066	< 0.001	Referent	1.0	–
Neglectful	234 (9.2%)	1092 (42.9%)	826 (32.4%)	394 (15.5%)			−0.293	0.746 (0.679–0.820)	< 0.001
Permissive	237 (1.3%)	5182 (29.3%)	7983 (45.1%)	4304 (24.3%)			0.361	1.434 (1.348–1.526)	< 0.001
Democratic	156 (1.4%)	3281 (28.4%)	6007 (52.0%)	2107 (18.2%)			−0.244	0.784 (0.738–0.832)	< 0.001
Living with parents before the age of 10 years									
Yes	748 (2.2%)	10,945 (31.7%)	13,526 (39.1%)	9362 (27.1%)	783.037	< 0.001	Referent	1.0	–
No	774 (3.0%)	9049 (35.3%)	11,317 (44.2%)	4478 (17.5%)			0.077	1.080 (1.027–1.135)	0.003
Number of close friends									
None	88 (1.4%)	1810 (29.2%)	2474 (39.9%)	1827 (29.5%)	400.275	< 0.001	Referent	1.0	–
1–2	375 (1.7%)	7434 (34.1%)	8719 (40.0%)	5263 (24.2%)			–	–	> 0.05
> 3	1059 (3.3%)	10,750 (33.4%)	13,650 (42.4%)	6750 (21.0%)			−0.480	0.619 (0.593–0.646)	< 0.001
The collective atmosphere in work/school									
Peace and tranquility	428 (2.2%)	4964 (25.9%)	9005 (47.1%)	4738 (24.8%)	1999.006	< 0.001	Referent	1.0	–
Occasional quarrels	660 (2.0%)	13,455 (40.1%)	12,514 (37.3%)	6941 (20.7%)			−0.222	0.801 (0.766–0.837)	< 0.001
Frequent quarrels	434 (5.8%)	1575 (21.0%)	3324 (44.4%)	2161 (28.8%)			0.162	1.175 (1.090–1.268)	< 0.001
The management style of work/school									
Autocratic	1180 (2.6%)	14,087 (31.1%)	19,777 (43.7%)	10,226 (22.6%)	1151.705	< 0.001	Referent	1.0	–
Laissez-faire	7 (0.3%)	1450 (61.7%)	469 (20.0%)	423 (18.0%)			–	–	> 0.05
Democratic	335 (2.7%)	4457 (35.4%)	4597 (36.5%)	3191 (25.4%)			−0.073	0.930 (0.880–0.983)	0.010
Marital status									
Unmarried	232 (1.9%)	4422 (35.3%)	5210 (41.6%)	2666 (21.3%)	1167.223	< 0.001	Referent	1.0	–
Married	766 (2.6%)	10,175 (34.7%)	11,779 (40.2%)	6579 (22.5%)			0.251	1.285 (1.221–1.353)	< 0.001
Divorced	295 (2.8%)	2294 (21.5%)	5210 (48.9%)	2853 (26.8%)			0.977	2.656 (2.491–2.833)	< 0.001
Remarried	222 (3.5%)	2669 (42.6%)	1922 (30.7%)	1452 (23.2%)			0.402	1.495 (1.384–1.615)	< 0.001
Widowed	7 (0.5%)	434 (29.9%)	722 (49.7%)	290 (20.0%)			0.772	2.164 (1.870–2.503)	< 0.001
Social activities of last 2 weeks									
0–2	803 (3.5%)	6783 (29.8%)	9699 (42.6%)	5505 (24.2%)	2363.756	< 0.001	Referent	1.0	–
3–5	239 (1.4%)	4483 (26.4%)	7459 (43.9%)	4822 (28.4%)			0.835	2.304 (2.183–2.433)	< 0.001
6–8	66 (0.8%)	2934 (35.1%)	3919 (46.8%)	1447 (17.3%)			0.332	1.394 (1.305–1.488)	< 0.001
> 9	414 (3.4%)	5794 (48.1%)	3766 (31.3%)	2066 (17.2%)			−0.887	0.412 (0.388–0.438)	< 0.001
Negative attention bias	10.22 ± 3.44	11.61 ± 2.21	12.56 ± 3.45	14.01 ± 4.10	1725.451	< 0.001	0.104	1.110 (1.104–1.116)	< 0.001
Negative memory bias	11.36 ± 3.45	11.20 ± 2.13	12.36 ± 3.68	13.10 ± 3.27	1100.525	< 0.001	0.067	1.069 (1.063–1.075)	< 0.001
Rumination	5.62 ± 2.11	5.90 ± 1.91	6.99 ± 2.47	7.65 ± 2.74	1732.229	< 0.001	0.126	1.134 (1.126–1.143)	< 0.001
Confirmed growth rate	0.42 ± 0.00	0.42 ± 0.08	0.66 ± 0.16	0.43 ± 0.15	14,648.272	< 0.001	–	–	> 0.05
Death growth rate	1.60 ± 0.00	2.46 ± 1.75	2.78 ± 1.06	2.79 ± 1.87	454.870	< 0.001	0.133	1.142 (1.129–1.155)	< 0.001

## Discussion

Our web-based cross-sectional study identified a significantly high prevalence of anxious and depressive symptoms in Chinese residents during the COVID-19 outbreak. China Mental Health Survey (CMHS) conducted a cross-sectional epidemiological survey in 2019, which revealed that 3.6% of Chinese adults had depressive disorder symptoms, while 5.0% had anxiety [30]. The current study suggested that depression and anxiety symptoms rapidly increased when a major infectious disease occurred. Chinese residents showed a higher level of anxiety than depression; these factors were positively correlated. These findings were consistent with the previous study, which suggested that individuals with anxiety were prone to depression, and depressed people tend to be anxious [31]. Owing to the isolation policy, the social activities of the residents were markedly reduced. The lack of social activities led to a higher level of anxiety and depressive symptoms. Social activity is an essential way of sharing interest and socializing with familiar people. Frequent participation in social activities has been proven to be associated with decreased depressive symptoms [32].

The death growth rate and the confirmed growth rate of COVID-19 reflect the severity of the epidemic. During our survey, the volatility trends of death growth rate and confirmed growth rate were not identical. Strikingly, the confirmed growth rate had a greater effect on depression severity, and only the death growth rate could positively affect the anxiety levels. During the pandemic of COVID-19, people could seek information from official channels to stay informed about the situation. Due to the transmission of COVID-19 and the continuous adjustment of diagnosis and treatment programs, the fluctuation of epidemic data may cause fluctuations in the public’s psychological pressure.

Male respondents showed a significantly lower level of depressive and anxiety symptoms than their female counterparts. This phenomenon is consistent with previous research, which concluded that women are much more susceptible to stress and more likely to develop higher psychological distress during the COVID-19 outbreak than men [33]. This may be explained by the fact that women have the social role of the lead caregivers in their family and occupational environments, making them more sensitive to stress than their male counterparts; however, this premise needs to be further researched. Additionally, individuals between 56- and 65-years-old presented more severe depressive and anxiety symptoms than other age groups. Studies and reports showed a relatively low incidence risk of COVID-19 for young people but a very high mortality risk for elders [34, 35].

The results also showed that education level and occupation were related to depression. People with a high school diploma were prone to develop severe depressive and anxiety symptoms compared to others. The education level may be related to the acquisition and identification of epidemic information; yet, this needs to be further investigated.

During the outbreak, the medical staff had a higher level of depression and anxiety than individuals from other occupational sectors because they had to face a heavier workload and were at direct infection contact with patients infected with COVID-19. These pressures could be internalized, resulting in depression and anxiety [36]. Collective atmosphere and management style are critical factors that affect mood at work or school. Our results showed that occasional quarrels were more effective than peace, tranquility, and frequent quarrels in decreasing anxiety, while democratic management was the best approach for reducing anxiety and depression. An egalitarian and relatively free working atmosphere can increase people’s enthusiasm for work and help to cope with stress. In contrast, a bad work atmosphere is related to depression and anxiety in the working population [37]. Also, friendship is a protective factor for mental health when facing stress [38], which can decrease anxiety levels.

The family was another aspect that affects anxiety and depressive symptoms during the COVID-19 outbreak. Living with two parents led to lower depression than living with other groups. Having sisters or brothers and not being the only child in the family could decrease anxiety. The democratic parenting style and living with parents until the age of 10 years of age were also conducive to the epidemic’s response. Good family functionality was negatively correlated to stress and depressive symptoms [39]. Marital status also reflected the family situation. Compared to unmarried people, divorced people were more likely to be depressive. Intriguingly, job, and income were rapidly reduced while marital conflicts increased during the isolation period, which may be associated with the risk of depression [40].

Negative cognitive processing bias, especially negative attention bias and rumination, had a negative role in regulating people’s mental health. People with negative attention, tending to reports such as death and confirmed cases, could easily succumb to negative stimuli. Lacking attention flexibility that engages with positive information and disengages from salient negative information might cause the failure of adaptive emotion regulation processes [41]. The rumination refers to the repeated thinking and analysis of negative emotions and feelings, which affects the onset of depression [42]. Individuals with ruminant traits are likely to be immersed in depression and anxiety and cannot extricate themselves because of the overwhelming news reports that have already created a repressive emotional atmosphere. Negative memory bias means a tendency to recall over-general memory and more negative memory than normal subjects, which has been regarded as an important risk factor for the emotional disorder [43]. People with this bias might recall negative information about a similar situation during the SARS epidemic. Abstract memory lacks concrete detail and tends to classify coronavirus as a disaster, thus resulting in enhanced anxiety. Individuals with negative cognitive processing bias tend to pay more attention to negative information, continue to ruminate on negative emotions, and make negative explanations for events’ results, thereby affecting their mental health [17].

Since January 2020, the National Health Commission of China has published several guidelines for emergency psychological crisis intervention, established psychological assistance hotlines, and providing online mental health education for the COVID-19 epidemic [44, 45]. All these measures contributed to easing the public psychological disturbance and psychological harm. Based on the findings of the current study, we generated the following recommendations: (1) focus should be placed on the vulnerable groups, such as the elderly, women, medical staff, and high school educated people; the focus should also be placed on people’s family situation and social activity; (2) provide information on COVID-19 prevention, treatment, control for the public, avoid the release of false information, and the spread of rumors; (3) use cognitive training to reduce mental distress, focus more on individuals with high negative cognitive processing bias, encourage and teach them to use emotion regulation strategies, and separate attention from negative emotions when they feel anxious or depressive, in order to maintain and promote their mental health; (4) provide authoritative psychological evaluation procedures and online psychotherapy to prevent further mental health problems.

The present study has several limitations. First, this study used the method of cross-sectional design, this it is not possible to make causal inferences. Second, the study was limited to an online survey, which may lead to selection bias. We might overestimate the ratios of anxiety and depressive symptoms because people who voluntarily choose to participate in the survey might be more aware of their mental health issues than those who did not participate. Third, due to the web-based study design, we could not control the origin of the participants’ regions and guarantee the sample’s representativeness, which might affect the research results.

## Conclusions

In this study, we assessed Chinese residents’ mental health status during the COVID-19 outbreak and identified the related risk factors for anxiety and depressive symptoms. The elderly, females, medical staff, and people with high school education were at higher risk of developing psychological issues. Negative cognitive processing bias had a negative role in regulating mental health. Psychological interventions should focus on vulnerable groups. Moreover, cognitive training that focuses on reducing negative cognitive processing bias might help alleviate the general public’s mental stress during the COVID-19 pandemic.

## Data Availability

The datasets used and analyzed in this study are available from the corresponding author on reasonable request.

## Abbreviations

SDS: Self-rating depression scale
SAI: State anxiety inventory
SARS: Severe acute respiratory syndrome
MERS: Middle East respiratory syndrome
NCPBQ: Negative cognitive processing bias questionnaire
